# Dynamic Hand Gesture Recognition Using 3DCNN and LSTM with FSM Context-Aware Model

**DOI:** 10.3390/s19245429

**Published:** 2019-12-09

**Authors:** Noorkholis Luthfil Hakim, Timothy K. Shih, Sandeli Priyanwada Kasthuri Arachchi, Wisnu Aditya, Yi-Cheng Chen, Chih-Yang Lin

**Affiliations:** 1 Department of Computer Science and Information Engineering, National Central University, Taoyuan 32001, Taiwan; koliskol@gmail.com (N.L.H.); sandelik@gmail.com (S.P.K.A.); wisnuadity@gmail.com (W.A.); 2 Department of Information Management, National Central University, Taoyuan 32001, Taiwan; ycchen@mgt.ncu.edu.tw; 3 Department of Electrical Engineering, Yuan Ze University, Taoyuan 32003, Taiwan; andrewlin@saturn.yzu.edu.tw

**Keywords:** hand gesture recognition, deep learning, multimodal system, context-aware

## Abstract

With the recent growth of Smart TV technology, the demand for unique and beneficial applications motivates the study of a unique gesture-based system for a smart TV-like environment. Combining movie recommendation, social media platform, call a friend application, weather updates, chatting app, and tourism platform into a single system regulated by natural-like gesture controller is proposed to allow the ease of use and natural interaction. Gesture recognition problem solving was designed through 24 gestures of 13 static and 11 dynamic gestures that suit to the environment. Dataset of a sequence of RGB and depth images were collected, preprocessed, and trained in the proposed deep learning architecture. Combination of three-dimensional Convolutional Neural Network (3DCNN) followed by Long Short-Term Memory (LSTM) model was used to extract the spatio-temporal features. At the end of the classification, Finite State Machine (FSM) communicates the model to control the class decision results based on application context. The result suggested the combination data of depth and RGB to hold 97.8% of accuracy rate on eight selected gestures, while the FSM has improved the recognition rate from 89% to 91% in a real-time performance.

## 1. Introduction

Gestures are one of the most natural ways of physical body movement, which can involve fingers, hands, head, face, or body to interact with the environment and convey meaningful information. Besides, gesture recognition is the way of the machine to classify or translate the gestures produced by a human into some meaningful commands. However, when communicating with the computer, hand gestures are the most common and expressive way of interacting more naturally among the other gestures. In recent years, hand gesture recognition has inspired new technologies in the computer vision and pattern recognition fields, such as Virtual reality [1,2] and Smart TV or interactive system [3,4]. Significant progress of this field has been accomplished in many applications, i.e., sign language recognition [5,6], robot control [7,8], virtual musical instrument performance [9,10].

Albeit the progressive efforts, fundamental problems in real-time usage persist, such as slow and expensive computation. Previous studies have been proposed to answer the challenges employing different methods such as devices-related method and glove based approach [11,12]. Even though most of such glove-based systems focusing on sensors, these external sensors enable to observe the user’s hand always. To address this drawback, a glove-based concept which utilizes the data gloves for human-computer interaction has proposed [13]. Besides the study [14] evaluate the performance of a wearable gesture recognition system that captures hand, finger and arm. Apart from devices and glove based methods, there are several handcrafted feature methods [15,16] and deep learning based methods [17,18] as well. Among them, deep learning model has deemed to solve the recognition and classification problems efficiently and accurately, yet the implementation in real-time application situations are limited. Hence, this study aims to introduce a hand gesture recognition system that works on a real-time application situation for Smart-TV like environment.

The proposed work combines 3D convolution neural network (3DCNN) followed by long short-term memory (LSTM) as a feature extraction model. Multimodal data, RGB and Depth data are incorporated as the input model. The Finite State Machine (FSM) control is used to restrict some gesture flows and to limit the recognition classes. Usage of small classes tends to showcase higher accuracy to that of big classes. The reuse of gestures for multiple commands in one application depends on the context of each application itself. Within the system, the global side of the hand gesture recognition is explored. Hindered by the range of action, instead of using finger feature for classification, the whole handshape as data input is implemented. Attention is focused on the hand by removing the background and unnecessary pixels. This approach allows the system to catch up in any variety of situation, e.g., crowded places, and may speed up the model computation.

In summary, this study considers the multiple sequences of input data and thus, the problem of solving the gesture recognition with sequence data is presented. Then it shows how to train only using a small set of the dataset in the deep learning-based model and the way of creating a small-size model that able to run smoothly in a real-time system and situation. In order to show the robustness of the proposed model, two kinds of applications were built. The first application is utilized to test the model accuracy, which consists of a simple interface showing the recognition result in a real-time situation. The second application is the complete application with the standard interface consist of 6 sub-applications. FSM control is implemented in the latter application to allow reusing the gesture according to the context of each sub-application. Twenty-four isolated gestures containing 13 dynamic and 11 static gestures are introduced to support the testing system. Twenty individuals’ data with five different environments and varying lighting conditions were collected as the dataset for training, validating and testing purposes. The performance of the dataset of eight selected gestures with different settings deduces accuracy higher than 90% in offline and real-time testing.

The rest of the paper is organized as follows. Section 2 reviews related work on gesture recognition problems and Section 3 explains the proposed method in detail and its combination with the FSM model. Next Section 4 discusses the experimental results and Section 5 concludes the work with future directions.

## 2. Related Work

Researches on gesture recognition problem have been growing in recent years. Started with the devices-based or glove-based techniques that capable of recognizing the gestures but suffer in the cost of production and a real-time situation, the works evolved to low-cost devices vision-based method, e.g., camera. The approach relies on the image and video processing technology to recognize the gestures translation. In the term of feature level, they can be divided into several levels: global hand feature level, finger feature level, 3D feature, skeleton feature, and trajectory of motion. Combining two or more features have been implemented by previous studies to enhance the accuracy result. Ren et al. [19] utilized distance matrix called FEMD (Finger-Earth Movers Distance) to extract the finger level features. Besides, further improvement of this work using the K-curvature algorithm was able to locate the finger positions [20]. The work of Li [21] suggested the Graham Scan algorithm for generating the convex hulls of finger detection. While owning many advantages, finger level is difficult to extract and often lead to reducing the speed of the system with the tradeoff on higher accuracy [22]. The trajectory level of features also works well on solving the gesture recognition problem in the term of Dynamic gesture recognition. The approaches, such as FSM [23], DTW [24], and HMM [25] are popular among the many methods. Since these methodologies witness the gesture in terms of trajectory, promoting the simplicity and robust approaches, but some gestures are unrecognizable in the temporal level of features. To overcome this issue, recognize gestures using the hand-crafted feature extraction method were proposed [26,27]. However, they are yet suffered from the lighting condition problem that may lead to reducing recognition accuracy. With the arrival of depth devices, e.g., Kinect or Intel Real Sense camera, such problems are not trivial anymore. The works [28,29,30,31] were successfully implementing RGB-D input combination to recognize gestures.

The aforementioned works are generally called hand-crafted features extraction or traditional method. While those methods are robust, but they are challenging to generalize the model for many cases. Some deep learning based approaches have been irrupted to achieve better results and mostly outperforming the “handcrafted” state of the art methods, to bridge such [32,33,34]. Inspired by such models, this study adopts long short-term memory (LSTM) model to solve the problem of long and complicated sequence in dynamic gestures problem. LSTM has become an important part of deep learning models for image sequence modeling for human action/gesture recognition [35,36]. The enhanced methods, such as Bidirectional RNN [37], hierarchical RNN [38], and Differential RNN (D-RNN) [39] were proven in recognizing the human actions and gesture recognition problems. Besides, the Convolutional Neural Network (CNN) in image classification problems have been successfully implemented in hand gesture recognition problems [40,41]. The proposed data augmentation strategies prevent CNN from overfitting when training the datasets containing limited diversity in sequence data. In some cases, using only temporal or spatial features are not enough to solve the hand gesture recognition problem. Thus, work using two-stream inputs, or fusion between spatial and temporal-based method are used. Two-stream convolutional network [42] learns spatial and temporal features separately. In addition, Long-term Recurrent Convolutional Networks (LRCN) model is capable of extracting such features. Moreover, 3DCNN which extract spatiotemporal features is superior in such tasks, since it uses the strong point of CNN on classify images and combine them with temporal features. However, it is limited to learn the long-term temporal information essential for hand gesture recognition. The work of Molchanov et al. [43] proposed the combination of using 3DCNN and RNN, fully connected spatiotemporal features transferred into RNN. Nevertheless, the spatial correlation information was lost in the RNN stage. Thus, this study proposed a new work to combine the 3DCNN and LSTM to solve the gesture recognition problem.

Gestures can also be considered as a finite sequence of states in the spatio-temporal model space in the FSM method. Several methods to recognize human hand gestures using an FSM model-based approach have discussed in previous studies [44,45,46]. Under the study of Hong et al. [46], each gesture has defined to be an ordered sequence of states using spatial clustering and temporal alignment. The spatial information is learned from the training images of gestures. The information acts as input to build FSMs corresponding model for each gesture. The FSM is used to recognize gestures from an unknown input sequence. In [45], FSM motion profile model was built, that has five states, start (S), up (U), down (D), left (L) and right (L) command corresponded to each gesture. The continuous spatio-temporal motion gestures are collected to build such models. The data then segmented into subsequences along with a single direction correspondent to each state. The system is highly reconfigurable, and no training concept is required. The model serves as input to a robot programming language for generating machine-level instructions in order to mimic the intended operation of the corresponding gesture.

These works show that the Finite State Machine is one of the sophisticated methods that has been used for gesture recognition. One of it uses to model the trajectory of movements, for example, hand or finger to recognize the gestures [23,46]. These works give us the general idea if restriction or control on gestures in each state according to the model in the FSM combined with the deep learning approach of classification is approachable.

## 3. Proposed Model

**Figure 1:**
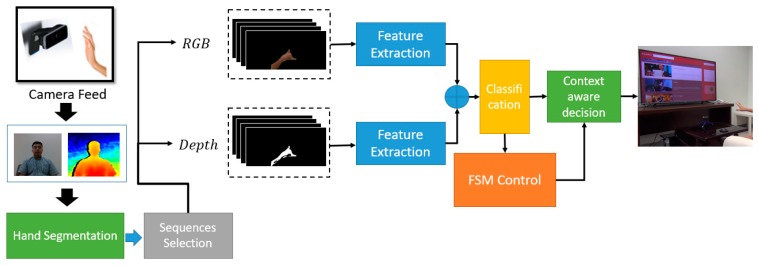
The overall architecture of the proposed system.

For the dynamic gesture recognition process, it is challenging to learn both spatial and temporal features with handcrafted feature extraction method, as mentioned by Wan et al. [47]. To address this challenge, we proposed a model, as seen in Figure 1. The proposed architecture consists of several processes such as data collection, data preprocessing, training, and testing model to achieve our purpose. Under this section, we explain all these processes of our proposed system in detail.

### 3.1. Data Collection

Ground truth creation is a fundamental issue in deep learning-based classification problems. Because of the absence of the standard gestures dataset suitable for a Smart-TV environment, self-defined gestures dataset was introduced. To create the dataset, we recorded the gestures from 20 individuals. All of these participants are right-handed and to reduce the bias and enhance the dataset, the user needs to follow several protocols. Before record gestures, the users divided into five groups, which include four people in each. Each recorded one gesture sequence of a particular user consists of a three-second length dynamic gesture which approximately contains 120 frames. The user needs to perform each gesture six times. It is not necessary always start the hand in the rest or in neutral position (hand down). When gestures are performing six times, each user needs to act differently, changing the speed and the position of the hand, in each attempt. Besides, the camera position, place and lighting conditions are different among each group.

**Figure 2:**
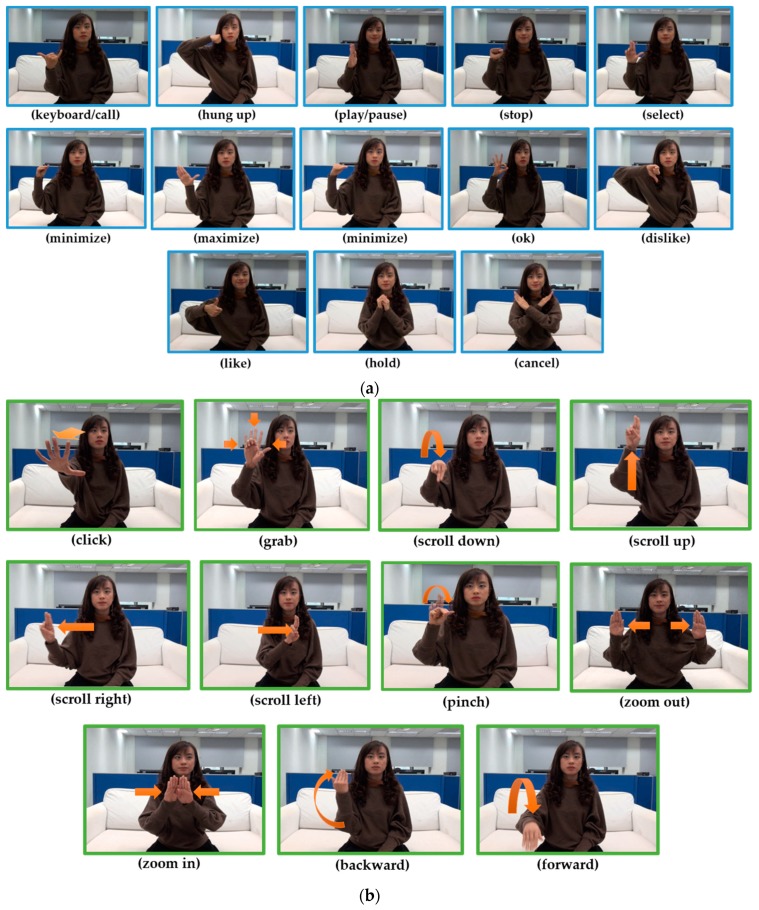
The 24 gestures designed and collected for Smart-TV like environment. For sample gesture videos of all gesture classes, please refer to our website available in http://video.minelab.tw/gesture/. (**a**) Thirteen static gestures; (**b**) Eleven dynamic gestures that visualize the directions of hand movements.

Originally 2880 videos recorded from 20 users when performing 24 gestures six times and sample images representing 24 gesture types are shown in Figure 2. As presents Figure 2a,b shows the 13 static and 11 dynamic gestures, respectively. However, we noticed some corrupted videos while creating the hand gesture dataset by manually filtering. To do a better classification, we removed corrupted videos before doing the pre-processing. The corrupted data is not only from specific persons, and hence those data consist of different users’ recordings. Therefore, our finalized hand gesture dataset consists of 2162 sequences (or videos) from 20 individuals.

A simple data collection tool is implemented to speed up the data collecting process and the collected data consist of sequences of gestures performed by real human actions. In addition, the gestures are recorded in five different environments: room with white background in dim light, a room with bright light and noisy background, outside the room (but still inside a building) near a window with bright light from the sun, outside the room far from the window with the soft sunlight, and inside the room that similar to home environment situation. The Real Sense SR300 depth camera as the primary tool for recording data was used to input the modality data into the proposed model. The recorded modality data include the RGB and Depth data. Since hand was considered as the main part of the gesture, part of the user’s body, including face and hand, was recorded. During the preprocessing step, the hand was extracted from the body as input to the model.

### 3.2. Data Preprocessing

Data collected in the recording section tend to have noise and unnecessary pixels. Therefore, cleaning data is an essential task to create accurate ground truth data. During the hand gesture recognition, the focus was given on the movement of the hand instead of other objects. Thus, the hand Region of Interest (ROI) was extracted from the given original pixel input. The model aims to focus its attention in the hand pixel instead of the other unnecessary points either from RGB image or Depth images. Under the experimental and discussion section, usage of the entire pixels for the input suggests the less effective way on gesture recognition problem in a real-time situation.

To extract the hand, given the whole RGB image $I_{r},$ and depth image $I_{d},$ fixed distance $d_{t}$ to remove the long distance background and minimum distance min of $I_{d}$ as the range filter was defined. Let $I_{rb}$ be the RGB image and $I_{db}$ be Depth image, after background removal. Based on the range $[min,\;d_{t}$] the average distance of a point $d_{av}$ in $I_{db}$ can be calculated as follows:

(1) $$\;d_{av}=\;\frac{\sum_{i}^{n}I_{db}^{i}}{n},\;\mathrm{where},\;I_{db}^{i}>\min\mathrm{and}\;I_{db}^{i}<\;d_{t}\;$$

Moving further, $d_{av}$ is used as a max filter to obtain $I_{rb}\;\;$’ and $I_{db}\;\;$’ (e.g., keep hands only) by the conditions below.

(2) $$\;\;I_{db}’=\{\begin{array}{c} 0,\;\mathrm{if}\;I_{db}^{i}>d_{av,}\;\\ I_{db}^{i},\;\mathrm{Otherwise} \end{array},\;\mathrm{where}\;i=1,\ldots ,\;\left(w\times h\right)\;\mathrm{of}\;\;I_{db}\;\;$$

(3) $$I_{rb}’=\{\begin{array}{c} 0,\;\mathrm{if}\;I_{rb}^{i}>d_{av,}\;\\ I_{rb}^{i},\;\mathrm{Otherwise} \end{array},\;\mathrm{where}\;i=1,\ldots ,\;\left(w\times h\right)\;\mathrm{of}\;\;I_{rb}\;\;$$

To keep the model attention to focus more on the hand with the detection of starting-ending points of the hand gesture, the predefined trigger box, Bt was used and crop the both $\;I_{rb}$’ and $I_{db}$’ according to the width $\left(w_{bt}\right)$ and height ($h_{bt}$) of the Bt, by the equation below to get the $I_{rb}^{cr}$ and $I_{db}^{cr}$ image.

(4) $$\;I_{rb}^{cr}=crop\left(I_{rb}’,\;w_{bt},\;h_{bt}\right)$$

(5) $$\;\;I_{db}^{cr}=crop\left(I_{db}’,\;w_{bt},\;h_{bt}\right)\;$$

Additional skin color filter added to remove more noise using the method by Kovac et al. [48]

(6) $$I_{h}=\mathrm{Skincolor}\left(I_{rb}^{cr}\right),\;I_{h}=[I_{h}^{RGB},\;I_{h}^{Depth}],$$

where (*R*, *G*, *B*) is classified as skin if: *R* > 95 and *G* > 40 and *B* > 20 and max{*R*,*G*,*B*}−min{*R*,*G*,*B*} > 15 and |*R*−*G*| > 15 and *R* > *G* and *R* > *B*.

**Figure 3:**
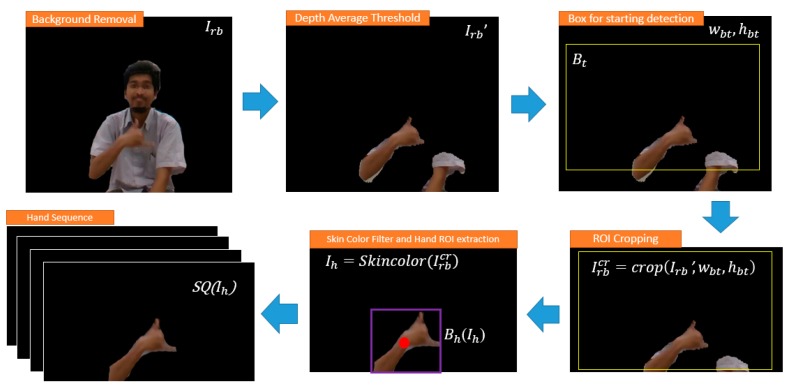
The preprocessing step of hand extraction based on the average depth threshold.

Given the RGB image $\;I_{h}^{RGB}$ after skin color process, contour extraction is performed on the image using the chain code of Open CV library to get the middle position of the hand. Prior to that, the Gaussian blur, dilation erosion, and edge detection were applied to create the hand image even clear. By using this middle position of the contour of the hand, the hand ROI $B_{h}(I_{h})$ was set to get the size area and position of the hand. Then, these two parameters are used as the starting-ending parameter decision to detect the necessary sequence to process. If starting detected, the system begins to collect the sequence of $I_{h}$ images consist of RGB data $I_{h}^{RGB}$ and Depth data $I_{h}^{Depth}$ in the form of $SQ\left(I_{h}\right)$. The preprocessing step of hand extraction based on the average depth threshold is seen in Figure 3.

Since dynamic hand gesture recognition is a sequence related problem, each person has different speed and starting point when performing gestures. Therefore, each image was considered and the number of sequences was aligned. Unnecessary gestures in the sequence were removed to allow faster processing. When a person performs a gesture, there are three main actions: the preparation, the nucleus, and the retraction. To align the sequence, first detect the preparation event of the user by tracking the middle position of the contour of the hand $\;h_{mid}$ until it touches the trigger box when performing the gesture. In order to get the starting gesture event or preparation event, the human habit of raising the hand around his face position when performed a gesture was used. Therefore, when setting up the trigger box around the face position, the preparation action of the gesture can be detected correctly.

The captured of 32 frames after the preparation event was carried out as the nucleus and retraction action of the gesture were processed for training, validating and testing the model. Thirty-two frames of sequences have opted since, in the sample, a user tends to perform an average of 32 frames after the preparation action.

### 3.3. Data Sequence Alignment and Augmentation

Normalizing a gesture sequence is a fundamental step in this study. Different users may perform a gesture in a different speed, while neural network input should be the same. Two conditions seldom raised in collecting sequences of gestures. One is the length of the sequence FL is lower than the predefined fixed length FS, which we set as 32 frames. Another problem is the value of FL should be higher than FS value. FL is the frame length of each gesture after detecting the starting and ending by the previous method. To solve the sequences alignment problem, two methods were applied: padding (i.e., $pd\left(SQ\left(I_{h}\right)\right))$ and down-sampling $\left(i.e.,\;ds\left(SQ\left(I_{h}\right)\right)\right)$ as defining bellow.

(7) $$\;SQ{\left(\left(I_{h}\right)\right)}^{’}\;=\{\begin{array}{c} pd\left(\left(SQ\left(I_{h}\right)\right)\;\right),\;\;FL<FS\\ \left(SQ\left(I_{h}\right)\right),\;\;\;\;\;\;\;\;\;\;FL=FS\;\\ ds\left(\left(SQ\left(I_{h}\right)\right)\;\right),\;\;FL>FS \end{array}\;$$

Padding infers additional image and depth data on the given sequence $SQ\left(I_{h}\right)$ by the last frame data ${\left(I_{h}\right)}_{FL}$ until FL=FS . As for the down-sampling, Equation (8) was adopted to get the index number of data that can be used through FL, to FS ratio. Given one gesture sequence $SQ\left(I_{h}\right)$ the index of data ${\left(I_{h}\right)}_{k}$ where k=1,…, FS from ${\left(I_{h}\right)}_{i}$ where i=1,…, FL are calculated by the following Equation.

(8) $$\;{\left(I_{h}\right)}_{k}=\;\frac{FL}{FS}*i,\;\;\;\;SQ{\left(\left(I_{h}\right)\right)}^{’}\;\;=\left\{{\left(I_{h}\right)}_{1},{\left(I_{h}\right)}_{2},\ldots ,{\left(I_{h}\right)}_{FS}\;\right\}\;$$

Besides from aligned the sequence, variation in the scaling, rotating, and translating RGB and depth data was added for augmenting the dataset and enhance the data generalization. Since gesture recognition requires fast recognition, rescaling the original image size into 50 × 50 pixel was performed. This number works well in the training and testing real-time in the term of speed.

### 3.4. Spatio-Temporal Feature Learning

In recent time, artificial intelligence in the form of deep learning has been reported to enhance the traditional method in hand gesture recognition problems successfully. Many researchers used the well-known CNN model, which consists of a one-frame-classification method to solve the static gesture recognition problem. However, during this study, the use of the spatial feature that becomes the speciality of CNN is not enough since the quality of the image might be distorted by the distance of the camera and rescaling of lower pixels to speed up the recognition process. Hence, only using the shape of the hand will not able to recognize the gesture properly. The combination of spatial-temporal features was deemed the best solution, especially the dynamic gesture recognition problems.

In this work, an enhanced dimension of the CNN named 3DCNN was implemented. This method was able to extract the temporal features by keeping maintaining the spatial features of the images and has been used in action recognition and video classification field. One of the representatives of this algorithm is C3D. While this algorithm is capable of extracting the short-term temporal features from the data sequence, it only extracts the data in a short-term way. This infers the inability of 3DCNN to memorize the longer sequences very well.

Since most of the gestures tend to have 32–50 frame per gesture, this 3DCNN model might not able to learn it better. Thus, the need for another network to learn long-term temporal features is necessary. Combination of the 3DCNN algorithm with the LSTM network was proposed to help to learn the long-time temporal features. The LSTM is capable of learning long-term dependencies with its sophisticated structure, including input, output and forget gates that control the long-term learning of sequence patterns. These gates regulate by sigmoid function, which learns during the training process from where it is open and close. Below 9 to 14 equations explain the operations performed in LSTM unit.

(9) $$i_{t}=\sigma \left(x_{t}w_{xi}+h_{t-1}w_{hi}+c_{t-1}w_{ci}+w_{ibais}\right),$$

(10) $$\;\;f_{t}=\sigma \left(x_{t}w_{xf}+h_{t-1}w_{hf}+c_{t-1}w_{cf}+w_{fbais}\right),$$

(11) $$\;\;z_{t}=tanh\left(x_{t}w_{xz}+h_{t-1}w_{hz}+w_{zbais}\right),\;\;$$

(12) $$\;\;c_{t}=z_{t}⊗i_{t}+c_{t-1}⊗f_{t\;},$$

(13) $$\;\;o_{t}=\sigma \left(x_{t}w_{xo}+h_{t-1}w_{ho}+c_{t-1}w_{co}+w_{obais}\right),$$

(14) $$\;\;h_{t}=o_{t}⊗tanh\left(c_{t}\right),$$

where, $\;i_{t}\;$, $f_{t}\;$, $o_{t}\;$, $z_{t}$ represent the input gate, forget gate, output gate, and cell gate respectively. $c_{t}$ and $h_{t}$ are memory and output activation at time *t*. The Equations (10), (11), (13) and (14) are the formulas for forget, cell, output gates and hidden state.

### 3.5. Multimodal Architecture

**Figure 4:**
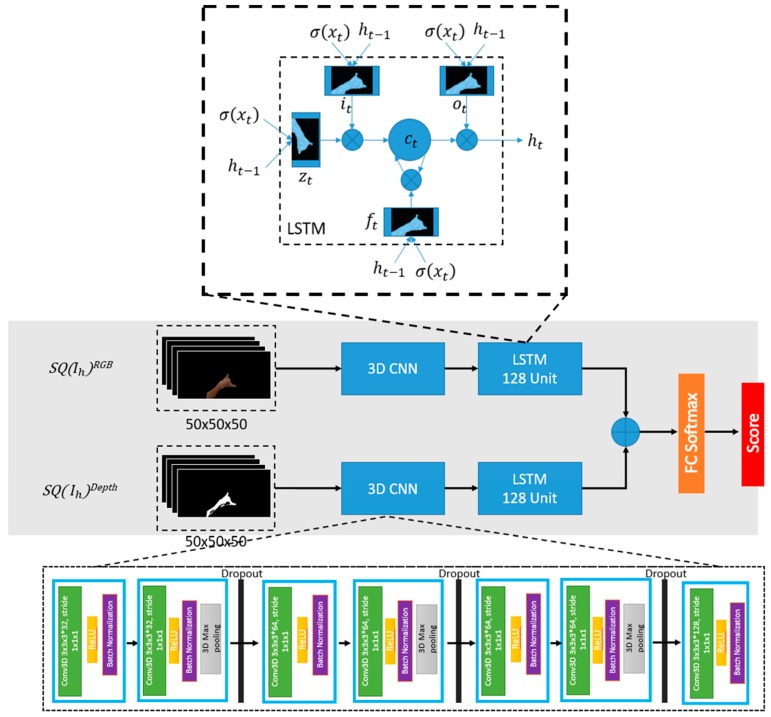
The proposed 3DCNN + LSTM architecture. The middle part of the figure shows the model’s general architecture and both bottom and top represent how the 3DCNN layer implemented and the way of LSTM unit works with the gesture sequence, respectively.

As shown in Figure 4, considering the above-discussed advantages of combining CNN and LSTM networks, the proposed multimodal architecture consist of 3DCNN layers, one stack LSTM layer and, a fully connected layer followed by the softmax layer. Batch normalization was utilized to allow the model to use much higher learning rates and less concerned about the initialization to accelerate the training process. The kernel size of each Conv3D layer is 3 × 3 × 3, the stride and padding are sizes of 1 × 1 × 1. The feature maps consist of four filter sizes such as 32, 64, 64 and 128, and double them up within each layer to increase the depth. Each Conv3D layer is followed by a batch normalization layer, a ReLU layer and a 3D Max Pooling layer with a pool size of 3 × 3 × 3. Features are extracted from the 3DCNN architecture then fed into the one stack of LSTM with 256 sizes of the unit. Several dropout layers were added in every section with the value of 0.5 and then computed the output probability result using the softmax layer.

**Figure 5:**
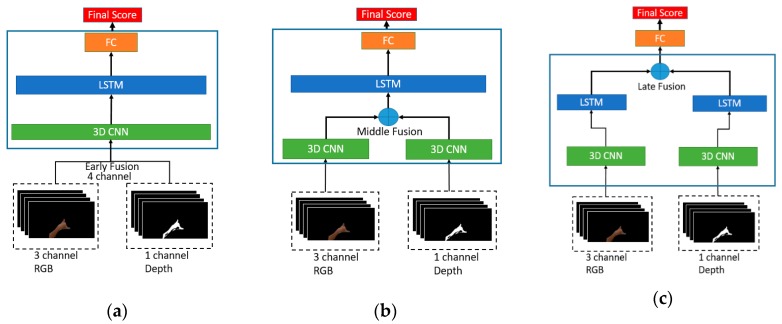
The proposed multimodal model architecture of 3DCNN + LSTM: (**a**) Early fusion multimodal; (**b**) Middle fusion multimodal; (**c**) Late fusion multimodal.

Using both depth and RGB as the input data might produce a better result rather than only using one stream input. Thus the multimodal model versions of the 3DCNN + LSTM are proposed. There are three kinds of multimodal types according to their fusion levels. The first way is called the early fusion model and this type only needs one stream of the model since they combine both input RGB and depth data into channels as shown in Figure 5a. Given $I_{i}$ RGB color image with three-channel and $D_{i}$ depth distance data with one channel, the new fusion input is the new image $ID_{i}$ with four-channel combinations. This way of fusion connects both RGB and depth data in a pixel-wise form. But, before putting the depth data into the 4th channel of $ID_{i}$ the normalization is necessary.

Since each person’s gestures in the dataset have a different distance to the camera, the normalization helps to generalize this difference. In the case of this work, normalize the depth data into the space of 0–255 color space is the best way. The second way is to combine the RGB and depth data by the middle fusion form as in Figure 5b. In this form, extraction of the feature of the image sequences was performed until the end of the 3DCNN layer in two separate way, then combine with the last layer before the LSTM layer. To fusion the features, there are several options. Concatenate, multiply, add, average, and subtract are the solution to fusion the features. In case of hand to hand only depth and RGB data were used for the proposed dataset, using multiply result the best compare to each other fusion method.

The 3rd fusion method is called the late fusion and, the architecture of the model is visualized in Figure 5c. The process of the combination is slightly similar to the middle fusion mechanism. Instead of combining the last layer of 3DCNN, the discrepancy lies on fusion at the end of the LSTM layer before doing the softmax. Thus both RGB and depth data will be trained in two separate models. The advantage of this fusion model is that different architecture parameters for different data input are permissible since it has a different model. Even though all of these fusion mechanisms train and test in the same parameters, this late fusion multimodal train slower than others. The comparison of all models discusses under Section 4.

### 3.6. Context-Aware Neural Network

One way to enhance the recognition rate in the real-time system is to let the model recognize smaller gesture class. In the system of real-time application, recognition class was limited in every context of the application. Hence, we can define this model as a Context-Aware recognition control system. In each context of an application, there are several sub-actions of a gesture that allows being performed or not. For example, in each application in one system, only five or fewer gestures were set to recognize. Not only this could promote the ease of the user to remember the gesture; it may enhance the recognition rate of the gesture recognition model. To do so, we use Finite State Machine (FSM) as a controller machine that can communicate with the Deep learning model by giving the restricted to the softmax decision probability by manipulating the weight in the last layer.

Generally, softmax uses as the output function of the last layer. The output of softmax is important because the purpose of the last layer is to turn the probabilistic score that sum to one. The softmax layer produced these probabilities that can be understood by humans from the given logits scores into values. When the input number of classes to the model is varying, it is indeed to modify weights of softmax accordingly since the output probabilities sum to one [49]. Within our context-aware network, each state function selects different gestures, and the state machine controls the context. First, the system in the current context or state communicates with FSM to get to know which gesture should be ignored or not. Then we apply the pre-defined weights to the last layer’s node that connects to the class which is ignored by the FSM. Therefore, only correct gestures are accepted, and the FSM can move to the next state.

### 3.7. FSM Controller Model

Let GRM be a Gesture Recognition Machine. GRM Takes current Camera Input, $ci_{x}\in \;CI$ and a current state $s_{i}\;\in \;S$, and output a classification result of the gesture $\;g_{j}\;\in \;G$. Where $G\;=\;\left\{g_{1},g_{2},\ldots ,g_{n}\right\}$ and $S\;=\;\left\{s_{1},\;\ldots \;s_{i},\;s_{i+1},\;\ldots ,\;s_{j},\;s_{j+1},\;\ldots ,s_{k},s_{k+1},\;\ldots ,s_{x}\right\},\;\;s_{x}$ is the exit state. The space of states S is divided into several subsets GRM: CI X S → G. For example $GRM\left(ci_{x},\;s_{12}\right)\;\to \;g_{8}$ (i.e., the machine takes a current camera input $\;ci_{x}$, in state $s_{12}$*,* the recognized gesture is $g_{8}$).

Let the FSM be a Finite State Machine that controls the context of gesture recognition system. The parameter of FSM are:

$FSM=\left(S,\;G,\;q_{0},\;q_{x},\;F\right)$, where, 

$S\;=\;S_{1}\;\cup^{\;}\;S_{2}\;\cup^{\;}\;S_{3}\;\cup^{\;}\;S_{4}\;\cup^{\;}\;\ldots \;\cup^{\;}\;S_{x}\;=\left\{\;s_{1},\;\ldots \;s_{i},\;s_{i+1},\;\ldots \;,\;s_{j},\;s_{j+1},\;\ldots \;,s_{k},s_{k+1},\;\ldots \;,s_{x}\right\}$ (i.e., S is divided)

$G=\left\{g_{1},\;g_{2},g_{3},\ldots ,g_{n}\right\}$ (i.e., n gestures) 

$q_{0}$*:*$s_{1}$ (i.e., initial sate) and $q_{x}$*:* $s_{x}$ (i.e., exit state)

F=S× G→ S (e.g., $F\left(s_{i},g_{j}\;\right)\to \;s_{j}$), the context dependent in next state function.

**Figure 6:**
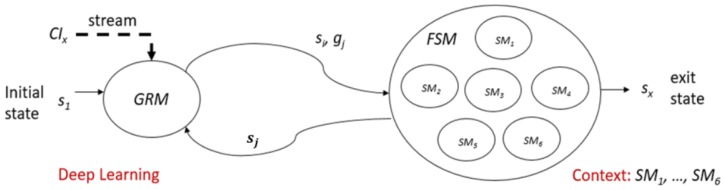
The scheme work of the FSM model controller with GRM (Gesture Recognition Machine).

**Figure 7:**
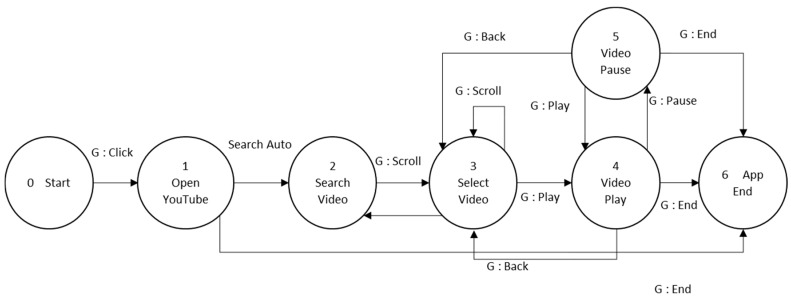
The FSM controller model of watch movie application.

Figure 6 gives a clear explanation of the FSM control section. For testing purpose, in our system, we have six kinds of applications: YouTube (Watch Movie) app, Facebook (Social media) app, Phone call app, Weather app, Chat room app, and Tourism app. Each application has a different set of FSM and a set of gestures to be used. Figure 7 is the example of one of the FSM system which is Watch Movie.

**Figure 8:**
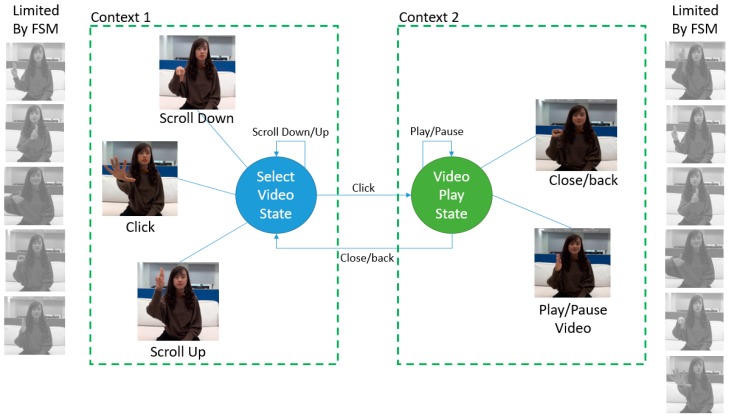
The Context-aware example of two contexts in the watch movie FSM model.

During the weight manipulating process, first, the FSM in the specific state returns the gesture that needs to focus on and to ignore. For example, the FSM model in Figure 7, six contexts represented by the node. For each node, there is a specific sub action or gesture restricted by the FSM. Thus, when playing with the video state, the gestures only able to use are “play/pause” and “back/close/end.” Figure 8 shows the illustration of how the context-aware works on gesture selection.

**Figure 9:**
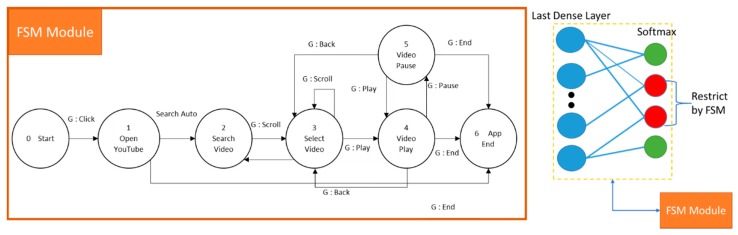
Example of FSM control of the deep learning model in softmax layer.

Manipulating on the weight of the last model layer was taken into consideration to ignore another gesture using the predefined weight limiter on those gestures. By doing so, the softmax layer will only focus on the rest of the gestures and show the result only those gesture that marked, as seen in Figure 9.

### 3.8. Training and Validating Strategies

For training and testing, 70% and 30% of data respectively used from our dataset and testing were done under two methods. The first method is offline testing, which uses three individuals’ data that not included in the train or validation set. The second is real-time testing that we suggest to an unknown user to test our application by performing some gestures.

The proposed model was implemented on the Tensor-flow platform and trained the model using ten mini-batches, until 50 epoch. Besides, we used Batch normalization to make the training process easier and faster, and the Adam and adaptive moment optimization with the parameters such as learning rate = 0.001, beta_1 = 0.9, beta_2 = 0.999, epsilon = 1 × 10^−8^, and decay = 0.0. The machine that the model trained was with the spec of Intel(R) Core(TM) i7-8700K CPU @ 3.70GHz, 32 GB of RAM, 24 GB of GPU NVIDIA GeForce GTX 1080.

## 4. Experimental Result and Discussion

**Figure 10:**
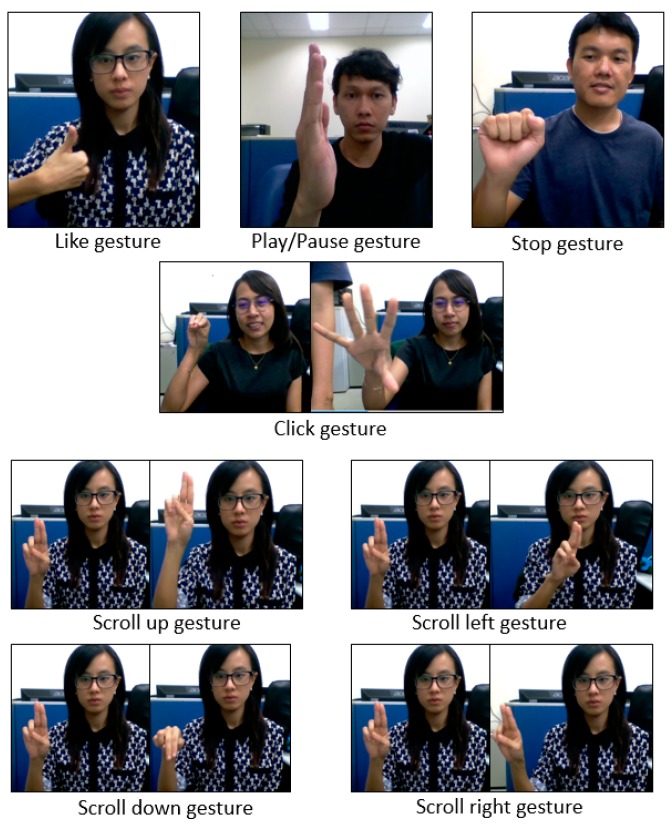
Eight gestures used in the real-time system. It includes three static and five dynamic gestures.

In order to test the proposed system in a real application, 8 gestures were selected from the 24 gestures dataset that collected previously. Those gestures selected based on the convenience uses in the application. The 8 gestures included 3 static and 5 dynamic gestures and Figure 9 represents the sample gesture types. The gestures as seen in Figure 10 are “like”, “play/Pause”, “stop” “click” “scroll up” “scroll down” “right swipe” and “left Swipe”.

### 4.1. Experimental Setup

Several experiments were conducted to test the model’s robustness. The first experiment is to check the importance of using hand focus attention on the input data to the proposed architecture. Using the same dataset, training and testing the model from several kinds of input data were carried out. In this section, the model in one stream input mode was tested. The input data types that we were the original RGB data without preprocessing and augmentation, RGB data with background subtraction on it and the RGB data that only focus to the hand. The second experiment is to compare the multimodal model with the one stream input model. Comparison between RGB data result of the first experiment and the second one was executed since the first experiment only conducts on one stream. To combine the proposed model’s different input streams, the late, middle and early fusion methods applied. The last experiment is the real-time experiment on two applications. One application has a simple interface without the FSM control, while the second application is in a complete application with a complex interface and FSM control inside it. People were asked to try the system, perform some gestures, and do some task in the application.

### 4.2. Comparison of Input Data Result

The first experiment meant to show the advantages of removal of the unnecessary pixels, such as background and focusing on one body part. As a result, in this case, the hand could increase the accuracy rate of hand gesture recognition. On top of that, alignment the hand sequence by detecting the preparation step of the hand gesture could also improve it as well. Table 1 shows the model trained using RGB images without any background removal and sequence alignment, and the results demonstrated that the average model accuracy falls to 73% under this setup. The second setup is based on the RGB with background subtraction and sequence alignment without focusing on the hand only. The half body was assigned with a black color background as the input. The result shows that a 90% accuracy rate of recognition was attained. The result demonstrated that removing the unnecessary part and detecting the hand preparation step as the sequence alignment improves the result a lot. Even though the result seems promising, when using in the real-time situation, the recognition rate falls below 70%, especially when a new person is using it. The reason is that the model learns the other part of the body as well, such as the face. Thus, during the third setup, we only used the hand information as the input data, including the sequence alignment. The result shows that a 94% accuracy rate on the test data without the data augmentation. Afterwards, the model was tested using depth data and the experimental result illustrates that only using depth data with only considering the hand cannot overcome the RGB setup data. The reason is that the designed gesture did not have a significant difference within the depth space. In the RGB space, the model could represent the shape clearly, comparing to the depth.

**Figure 11:**
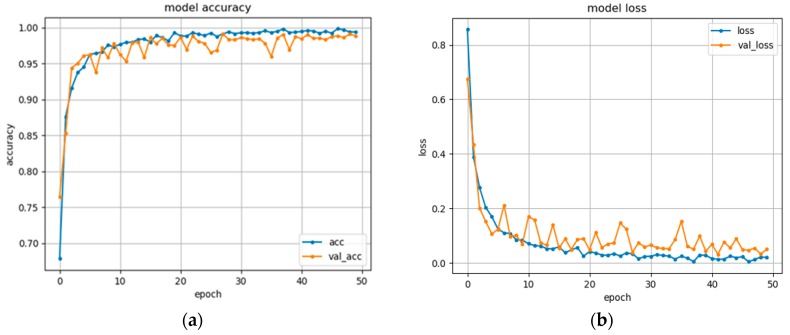
The performances of the late fusion 3DCNN+LSTM model with 8 gestures used in the system: (**a**) the model accuracy with the number of epochs; (**b**) the model loss against the number of epochs.

During this study, the data augmentation is done by scale, rotation and change the brightness of the dataset and the last setup is to proof the data augmentation’s advantage when increasing the accuracy rate. Within the data augmentation process, the amount of gestures increases from 2162 to 6486 sequences of the gestures. Figure 11a,b respectively show the training and validating accuracies and losses of our late fusion 3DCNN+LSTM model using RGB and depth data focusing on hand as the input with the augmented dataset. The results demonstrated that with our dataset, the model could reach the training and validation accuracy of 99.4% and 99.2% respectively.

### 4.3. Comparison of Multimodal Input Data Result

The next experiment is to test the robustness of the proposed model with different fusion benchmarks discussed under Section 3.5. Among the several conducted experiments, the first used the late fusion technique with the depth and RGB input. From the previous experiment, the best result of the model is using the hand-focusing input data with sequence alignment. Thus, the same setup was re-applied for RGB and hand focusing depth data. However, the second and third experiments also used the same setup but with early and middle fusion methods combining depth and RGB. The results illustrated in Table 2 shows that using late fusion method could achieve better performance compared to other fusion methods.

### 4.4. Real-Time Experimental Result

As illustrates in Table 3, during the Real-Time testing, average accuracy has down from 97.6% to 89%. We instructed seven people to perform under two types of conditions. The first condition is to let the user perform gestures one by one, and the user needs to perform each gesture 10 times. Under this condition, the user could perform well with 95% accuracy. The second condition is to let the user perform the gesture by him/herself in a sequence manner, and in this case, the accuracy started to go down to 93%. The reason to decrease accuracy is that the user has forgotten the next movement when transitioning from one gesture to another. The third real-time testing condition is to let the user use the real application. While using this application, the result down to 89% accuracy. These problems are due to several factors. Some user may felt uneasy and nervous when performing the gesture. In application, the user cannot witness him/her self or his/her hand movement. Furthermore, they did not memorize the gesture movement precisely, due to limited practice time of 5 min. The other problem may due to the FPS (Frame per Second) getting lower in the application since it contains many complicated functions. However, when tested using real complex application with FSM controller, the real-time testing accuracy increased from 89% to 91% because when a user performs awkward and no confidence. The FSM model could narrow the class to the smaller class according to the context, in this case, is the current application state.

### 4.5. Real-Time System

**Figure 12:**
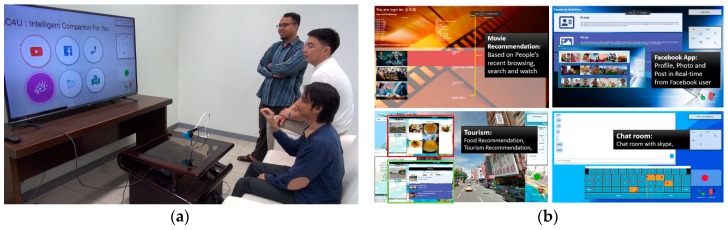
Showing the implementation of the proposed model in the Real-Time system: (**a**) People performing gestures to control the system; (**b**) The example content of the system. The following link is the system in action (http://video.minelab.tw/gesture/).

As mentioned earlier, to show the usability of our proposed method, the Real-time application system called the IC4You was built. The system is implemented with the gesture recognition system as the core of the interface controller. The IC4You system has been performed in several events within Taiwan, and already tested by various users. The demo event was conducted successfully and received much positive feedback to improve the system in future. Figure 12 visualizes the way of the system working in Real-Time with a smart TV-like environment. The project website available at http://video.minelab.tw/gesture/ provides how users perform gestures with a live demo of real-time testing.

## 5. Conclusions and Future Work

This paper presents the work to solve the gesture recognition problem on real-time application situation by combining RGB and depth modalities as the input for the deep learning model. The combination of 3DCNN and LSTM model could extract the spatio-temporal features of the gesture sequence, especially with the dynamic gesture recognition. In the term of using it in a real-time application, adding the FSM controller model could narrow the gesture classification search on the model into a smaller one that could ease the model work and enhance the accuracy result. Dataset collection of 24 gestures were designed associated with a smart TV-like environment to test the proposed model. As for the application’s real-time testing, eight gestures were to examine the robustness of our work. The result suggests if the FSM controller may enhance the accuracy result in real-time applications. For the future work, transfer learning is proposed by training the model with large datasets such as the Sports-1M dataset or ChaLearn gesture dataset in order to enhance the accuracy result. Also, we plan to report the comparison result with other similar work, only in terms of gesture recognition. Another possible future direction is adding the function for gesture modification for the user utilizing transfer learning. Moreover, the discussion of selection and usability design of the gestures, especially for Smart-TV environment, plan to conduct and report in the future as well.

## Figures and Tables

**Table 1:** The proposed model performance comparison with different input data setups.

No	Input Setup	Accuracy
1	3DCNN + LSTM + RAW RGB data without sequence alignment	73%
2	3DCNN + LSTM + RGB data with background removal + body + sequence alignment	90%
3	3DCNN + LSTM + RGB data only focusing on hand + sequence alignment	95.8%
4	3DCNN + LSTM + Depth data only focusing on hand + sequence alignment	94.4%

**Table 2:** Performance comparison of proposed 3DCNN+LSTM model with different fusion methods.

No	Input Setup	Accuracy Rate
1	Early Fusion + Depth + RGB hand only	95.8%
2	Middle Fusion + Depth + RGB hand only	95.1%
3	Late Fusion + Depth + RGB hand only	97.6%

**Table 3:** Comparison of the average accuracies of real-time testing with the proposed multimodal.

No	Input Setup	Accuracy
1	Using simple application with simple command gesture—Test 1	95%
2	Using simple application with sequence command gesture—Test 2	93%
3	Using real complex application with no FSM controller—Test 3	89%
4	Using real complex application with FSM controller—Test 4	91%
